# PRIORITIES ALIGNED DEPRESCRIBING FOR PERSONS LIVING WITH DEMENTIA AND THEIR CARE PARTNERS

**DOI:** 10.1093/geroni/igae098.0652

**Published:** 2024-12-31

**Authors:** Aanand Naik

**Affiliations:** The University of Texas Health Science Center, Houston, Texas, United States

## Abstract

PlwD average 8 daily medications compared to 3 for persons without dementia. PlwD have high polypharmacy burden from anticholinergic and psychotropic medications. PlwD, their care partners and clinicians endure the workload of managing multiple medications, and want to decrease this burden. Thus, a shift that reduces polypharmacy, and focuses medication use on what matters most is needed. Deprescribing is an approach to reduce polypharmacy for PlwD. However, deprescribing studies for PlwD have a narrow scope. Few studies place deprescribing within ambulatory care contexts for patients with mild to moderate dementia. Appropriate deprescribing in dementia requires identifying health priorities. Deprescribing is most appropriate when guided by priorities of patients and care partners. Patient Priorities Care (PPC) is an evidence-based approach to health priorities aligned decision making. We found evidence that PPC facilitates deprescribing by identifying health priorities that are misaligned with current medications. When used in the care of PlwD and their care partners, PPC could facilitate deprescribing. Less is known about how PPC works in the triadic context of clinician, care partner, and PlwD. Care partners may have concerns about stopping medications considered inappropriate such meds help manage symptoms. Clinicians may focus on cognitive and functional harms of medications without considering tradeoffs of stopping medications. Without eliciting priorities, families and care partners of PlwD may make decisions to stop medication without communication with their clinicians. We recently completed a study using PPC to deprescribe unnecessary medications among PlwD. We will discuss our findings and the implications for future work.

